# Evolution of cooperation and trust in an N-player social dilemma game with tags for migration decisions

**DOI:** 10.1098/rsos.212000

**Published:** 2022-05-11

**Authors:** Sandeep Dhakal, Raymond Chiong, Manuel Chica, The Anh Han

**Affiliations:** ^1^ School of Information and Physical Sciences, The University of Newcastle, Callaghan, New South Wales 2308, Australia; ^2^ Department of Computer Science and Artificial Intelligence, Andalusian Research Institute in Data Science and Computational Intelligence, DaSCI, University of Granada, 18071 Granada, Spain; ^3^ Department of Computing and Games, Teesside University, Middlesbrough, Tees Valley, UK

**Keywords:** evolution of cooperation, N-player games, migration, trust, tags

## Abstract

We present an evolutionary game model that integrates the concept of tags, trust and migration to study how trust in social and physical groups influence cooperation and migration decisions. All agents have a tag, and they gain or lose trust in other tags as they interact with other agents. This trust in different tags determines their trust in other players and groups. In contrast to other models in the literature, our model does not use tags to determine the cooperation/defection decisions of the agents, but rather their migration decisions. Agents decide whether to cooperate or defect based purely on social learning (i.e. imitation from others). Agents use information about tags and their trust in tags to determine how much they trust a particular group of agents and whether they want to migrate to that group. Comprehensive experiments show that the model can promote high levels of cooperation and trust under different game scenarios, and that curbing the migration decisions of agents can negatively impact both cooperation and trust in the system. We also observed that trust becomes scarce in the system as the diversity of tags increases. This work is one of the first to study the impact of tags on trust in the system and migration behaviour of the agents using evolutionary game theory.

## 1. Introduction

The evolution of unselfish behaviour in a population of selfish individuals has long been the subject of scientific studies in diverse fields, including social sciences, biology, economics and computer science [1]. Evolutionary game theory (EGT) is one of the most used theoretical frameworks in the literature for understanding how unselfish behaviour, such as cooperation and trustworthiness, emerges from the interaction of selfish individuals with bounded rationality, particularly in social dilemmas [2].

It can be difficult to build mutual trust among the members in a population because of the high cost of fully understanding and accurately knowing each other’s willingness to cooperate [3], and this can influence the evolution of cooperation [4,5]. Various biological and social mechanisms have been studied in the literature to remedy this, and one of the most popular is the tag-based mechanism. Tags or labels, which are externally perceptible features that are consistent among groups of animals or humans, can be used to determine appropriate response strategies in societies [6]. Tags also provide individuals with information about the characteristics of those belonging to different groups [7].

Prior studies in the game-theoretic literature have focused on using the tag mechanism to study if those strategies that determine their actions based on others’ tags (or labels) can promote and sustain cooperation in a variety of games. These studies have shown that such strategies can indeed promote cooperative behaviour in games, such as the iterated Prisoner’s Dilemma [8], Snowdrift [9], Commons Dilemma [10], among others. For example, with a tag-mediated altruism model, Hadzibeganovic *et al*. [11] showed that tags can be used as an indirect form of reciprocity, which can then promote cooperative behaviour. More recent examples of such mechanisms include simple cues such as smiles to embody information that influences pro-social behaviour in social interactions [12], a third party controller that observes the interactions and labels the agents as either cooperators or defectors [3], and a dynamic tag determined by the system age or strategy age [13].

It is also well known that models of cooperation in networks must combine strategy and structural changes [14]. Santos *et al.* [15] proposed a model of cooperation in networks that allowed agents to change both their behaviour and their connections with each other, thus mimicking the concept of migration. Following this, a number of studies have attempted to understand different migration topics such as risk-driven migration [16], opportunistic migration [17], expectation-driven migration [18], immigration and assimilation into the host country’s culture [19], voluntary participation [20] and spread of group beneficial cultural variants through migration [21] using EGT. Trust and trustworthiness within networks is another related concept that has become a popular research topic; several studies have attempted to study the dynamics of trust and trustworthiness using social dilemma game models [4,22–25].

There are, however, only a handful of studies in the literature that integrate the concepts of both tags and migration. In one such study, Hadzibeganovic & Xia [26] analysed the impact of tag-based interaction in mobile technological networks with variable resources, along with the evolution of four strategies in the population. Similarly, most studies employ tags to determine behaviour in terms of either cooperation or defection (e.g. [11,27]). Tags have not been used to direct and understand the migration decisions of agents in the population. Another gap in the literature is that most studies have employed dynamic tags, i.e. the tag of an agent can change during the simulation, which is akin to perceptions about individuals changing in the real world. However, in many scenarios, it is not possible to change the tags that the agents are assigned: for example, their physical attributes, ethnicity, language spoken, among others. Cohen & Haun [28], for instance, considered the plausibility of tag-based cooperation through linguistic cues such as accents.

In this study, we present a model that integrates the concept of tags—by assigning tags to agents in an N-player game, and migration. Instead of driving the cooperation/defection decisions of the individuals, the tags determine the migration decisions of individuals in the population. Agents in the model trust tags differently based on the results of their interactions with agents belonging to those tags. The model also includes two layers of social grouping; agents are part of a social network that they use for learning, and agents are also part of distinct groups that they can migrate to and from depending upon their game fortunes. As agents interact, they develop varying trust in the different tags [29]. How much agents trust different groups is determined by the composition of the groups in terms of those tags. This trust value is then used to make migration decisions.

We use the model to understand how the game evolves when considering the basic concepts of tags, and migration that is determined by trust in groups composed of agents with different tags. In particular, we attempt to answer the following questions:

— if tags and trust in those tags can impact the level of cooperation in the population without implicitly or explicitly directing tag-based behaviour among the agents,
— whether the population evolves such that different tags become more trusted than others, throughout the population,
— if tags congregate into different groups when allowing migration among different groups based on the trustworthiness of the group (determined by the composition of tags in the group),
— if migration based on tags and trust in those tags impacts the cooperation inclinations of the players.

The rest of this paper is organized as follows. The model is described in detail in §2. The experimental set-up and results are discussed in §3. We conclude the paper by presenting our conclusions and discussing some future research areas in §4.

## 2. Model

### 2.1. Game structure

The game is an N-player game comprising non-overlapping groups of agents that are also part of a social network. Agents can interact with their group members for making migration decisions, and interact with their connections in the social network for their social learning mechanism. Each agent *i* has a strategy *s*_*i*_ ∈ {*C*, *D*}, where *C* and *D* refer to cooperation and defection, respectively.

A group, *g*, is a collection of agents, which can represent a geographical entity. An agent belongs to only one group at any one time, but can migrate to a different group at the next timestep. All groups have the same number of agents at initialization, but their size, |*g*|, varies as agents migrate between groups during the simulation [30]. Group members influence the learning and payoff of their fellow group members. This division mimics the concept of metapopulation, in which the population is allocated into more than one spatially separate subpopulation [31].

Similarly, all agents in the model are part of a social network. The agents are the nodes of the social network and the edges represent their connections. All edges are bidirectional and the number of edges connected to each agent is dependent on the type of network. Agents use the social network for social learning during the evolution of the game. They socially adapt by selectively imitating the actions of their connections in the network (see §2.4 for further information). In contrast to the dynamic nature of the groups, the social network is static, i.e. the network and its connections remain constant throughout the simulation.

### 2.2. Trust dynamics of the game

Each agent *i* is assigned a tag, *τ*_*i*_, at the beginning of the simulation. The different tags in the system are fixed at the start of the simulation as $T=(t_{1},t_{2},\ldots ,t_{\Theta })$, where Θ is the number of tags in the game. Tags represent the concept of social categorization, which is a natural cognitive process that humans use to place individuals into social groups [32].


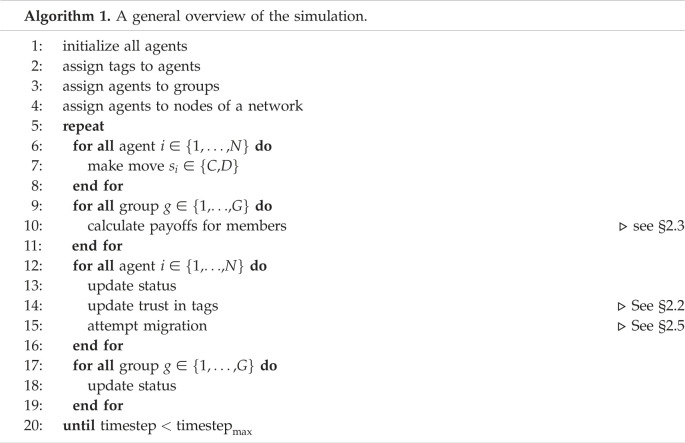


Agents do not keep track of their trust in each individual agent, but only their trust in the different tags in the game. Therefore, when deciding whether an agent, *i*, can be trusted, other agents will consider only their trust in its tag, *τ*_*i*_. As a result, an agent will have equal trust in all agents with the same tag.

The trust of a focal agent in each tag is updated at each timestep *t* based on the previous interactions of the focal agent’s interactions with other agents belonging to that tag. This is proportional to the payoff of the agent and the population of the different tags in the agent’s group. More specifically, the trust function of any agent *i* in tag *τ* at time *t* is calculated as [29]:

2.1 $$\phi_{i,\tau }^{t}=(1-\mu )\cdot \phi_{i,\tau }^{t-1}+\mu \cdot p_{i}^{t}\cdot \frac{\left|\tau_{g}^{t}\right|}{\left|g_{i}^{t}\right|},$$

where *μ* is the decay value in the range [0,1] that determines how much of the previous trust value is retained, $p_{i}^{t}$ is the payoff of agent *i* at timestep *t*, $\left|g_{i}^{t}\right|$ is the population of agent *i*’s group *g* at timestep *t*, and $\left|\tau_{g}^{t}\right|$ is the number of agents with tag *τ* in group *g* at timestep *t*.

An agent’s trust in a group, including its own group, is dependent on the composition of different tags in that group, and is calculated as follows:

2.2 $$\phi_{i,g}^{t}=\frac{\sum_{\tau \in T}|\tau_{g}^{t}|\cdot \phi_{i,\tau }^{t}}{\left|g^{t}\right|},$$

where *T* is the set of all tags, $\tau_{g}^{t}$ is the population of tag *τ* in group *g* at timestep *t*, $\phi_{i,\tau }^{t}$ is the trust of agent *i* in tag *τ* at timestep *t*, and |*g*^*t*^| is the population of group *g* at timestep *t*.

### 2.3. Payoff calculation

The collective benefits of the efforts put forth by all agents can be enjoyed by every agent regardless of its actions. The cost of cooperation is, however, paid only by the cooperators. The payoff, $p_{i}^{t}$, of focal agent *i* at timestep *t* is, therefore, calculated using a Fermi rule [33] as follows [34]:

2.3 $$p_{i}^{t}=\left\{ \begin{array}{cc} b\cdot |g_{C}^{t}|-\frac{c\cdot (|g^{t}|-1)}{|g_{C}^{t}|+1},\; & \;\text{for cooperators}\;\\ b\cdot |g_{C}^{t}|,\; & \;\text{for defectors},\; \end{array}\right.$$

where *b* and *c* are the benefit of cooperation (per agent) and the overall cost of cooperation, respectively; |*g*^*t*^| and $\left|g_{C}^{t}\right|$ are the group’s population and the number of total cooperators in the group, respectively, at timestep *t*. Values of the parameters for the game must fulfil *b* > *c* > 0. The dilemma strength [35,36] in the game is thus quantified by *r* = *c*/*b*. Increasing *c* and decreasing *b* lead to a stronger dilemma; conversely decreasing *c* and increasing *b* lead to a weaker dilemma.

However, there are situations in the real world where a minimum effort is required for some benefit to be achieved from the cooperative effort. An example is the case of flood protection where a minimum number of individuals are required to set up an artificial dam (for flood protection) [37]. If the required minimum number of individuals do not participate in the task, none of the agents (including the free-riders) receive any benefit. To this end, our model includes a threshold ratio, 0 < *m* ≤ 0.5, such that for a group of size |*g*|, any benefit is achieved only if the number of cooperators, |*gC*|, is at least ⌈ m⋅|g|⌉ . Now, an agent’s payoff, when the threshold is not met, is calculated as

2.4 $$p_{i}=\left\{ \begin{array}{cc} \frac{-c\;\cdot \;(|g|-1)}{\left\lceil \;m\;\cdot \;|g|\right\rceil }\; & \;\text{for cooperators},\;\\ 0\; & \;\text{for defectors}.\; \end{array}\right.$$

Even though all the groups initially have the same size, over the course of the simulation, the population of groups varies due to migration. Since an agent’s payoff is dependent on the number of agents it interacts with (in this case the group members), the absolute payoff is biased towards agents with a higher number of connections, i.e. agents that are members of larger groups [38]. Therefore, an agent’s payoff is normalized on a scale of 0 to 1 based on the minimum and maximum possible payoffs in the group as follows:

2.5 $$p_{i}=\frac{\;p_{i}-p_{\min}}{p_{\max}-p_{\min}},$$

where *p*_*i*_, *p*_min_ and *p*_max_ are the absolute, minimum and maximum possible payoff values in the group, respectively. In any group, the minimum possible payoff is obtained by an agent, if it is the only cooperator in the group. Similarly, the maximum possible payoff is obtained by an agent, if it is the only defector in the group.

### 2.4. Agent strategy update

At the end of each timestep, after all agents have calculated their payoffs, each agent has an opportunity to update its strategy based on its payoff and its neighbours’ payoffs in the social network. The strategy update follows an evolutionary procedure based on imitation of neighbours and can be interpreted as information exchange in a social learning process [39]. The agents’ actions follow a simple rule of conditional imitation: randomly pick a fellow group member or a connection in the social network, and conditionally imitate their last action (at timestep *t* − 1) in proportion to their payoff. The probability of imitating the randomly chosen agent is determined using the Fermi rule as follows:

2.6 $$\mu =\frac{1}{1+e^{-\beta (p_{o}^{t-1}-p_{i}^{t-1})}},$$

where *μ* is the probability of imitating the last action of the chosen agent, *β* is the background noise (set to 1 in this study), $p_{i}^{t-1}$ is the current agent’s normalized payoff, and $p_{o}^{t-1}$ is the other agent’s payoff at timestep *t* − 1.

### 2.5. Agent movement

Individual agents can migrate to another group from their current group to escape from untrustworthy neighbours/groups and to seek more trustworthy neighbours/groups. Migration decisions are made by individual agents at the end of each timestep. An agent considers migrating to another group if its trust in the current group is less than its *trust threshold*, $\hat{\phi }$, which is a value in the range [0,1] and is set once at the start of the simulation. $\hat{\phi }$ is the same for all agents. Based on the restrictions on minimum and maximum allowed group sizes (see §2.1), migration away from the group is also forbidden if the group only has the minimum required members, and in-migration is not allowed if the destination group already has the maximum allowed members. The destination group can be any group that has as a member any of the focal agent’s connections in the social network. This allows the population of groups to fluctuate during the simulation.

Some previous studies have demonstrated how different migration preferences can have varied influence on the evolution of cooperation [40]. Inspired by this, in this study, we use and compare three different methods for selecting the migration destination. These methods are described below:

*Random Destination Selection (RDS)*. In this method, a random destination is selected from the list of available destinations. Once a destination has been selected, the agent migrates to that destination if it is accepting new members.
*Proportional Group Trust based Selection (PGT)*. This is a more restrictive version of the Random Selection method. Once a potential destination is randomly selected, the focal agent *i* can move from its current group *g* to the destination group *g*′ with a probability, *ω*, dependent on the agent’s *trust* in both groups using a Fermi rule as follows:
  2.7 $$\omega =\frac{1}{1+e^{-\gamma (\phi_{i,g}-\phi_{i,g^{\prime }})}},$$
  where *γ* is the background noise (set to 1 in this study); and *ϕ*_*i*,*g*_ and *ϕ*_*i*,*g*′_ are agent *i*’s trust in its current group and the destination group, respectively.
*Most Trusted Group Selection (MTG)*. In this method, an agent selects the group it trusts the most from the list of available groups for migration. As discussed in §2.2 an agent’s trust in a group depends on the composition of different tags in that group and the agent’s level of trust in those tags (see equation (2.2)).

## 3. Analysis of the results

### 3.1. Experimental set-up

We conducted extensive simulations to study the relationship between cooperation, trust and migration, as well as the impact of trust thresholds, number of tags, and the number of groups on the level of cooperation, migration and trust. All experiments were repeated across the spectrum of cost-to-benefit ratio *r* ∈ [0 · · · 1]. The population of each group was restricted by 2 ≤ *n* ≤ 0.25 · *N*, where *N* is the population size. Setting the minimum size of a group to two ensures that each agent has at least one interaction partner. Similarly, the maximum group size restriction helps avoid the congregation of all agents in one group.

The trust threshold is defined from the set {0.1, 0.3, 0.5, 0.7}. The Barabasi–Albert preferential attachment algorithm [41] was used to implement the scale-free social network for the experiments. This algorithm adds new nodes to the network one at a time, and each new node is connected to *M* existing nodes with a probability that is proportional to the number of links the existing nodes already have. The higher the value of *M*, the higher the density of the network. In our model, *M* = 8. All simulations were run for 5000 timesteps, with a population of 1000 individual agents. Each simulation was repeated for 50 independent Monte Carlo (MC) realizations.

### 3.2. Cooperation, migration and trust for different *r* values

We conducted the first set of experiments with two tags, a cooperation threshold, $\hat{\theta }$, of 0.5, and four values of $\hat{\phi }$ (0.1, 0.3, 0.5, 0.7). The PGT method was used by all agents to select the migration destination. Our previous study had shown that high levels of cooperation threshold can promote cooperation in N*-*player social dilemma games [16]. Groups had an initial population of 10 agents, meaning that there were 100 groups throughout the population (since groups need to have at least two members).

**Figure 1.**
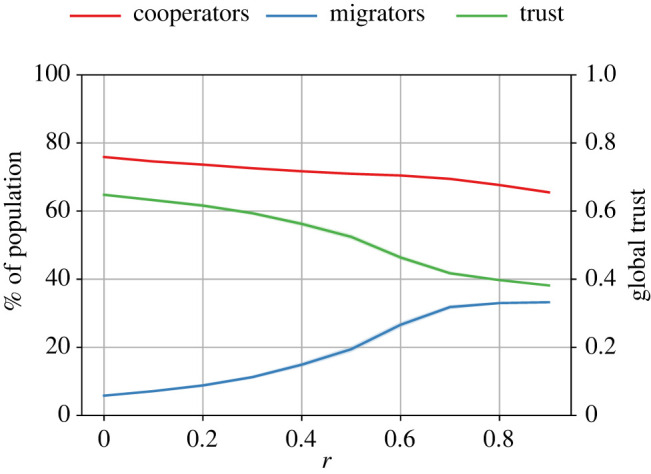
Levels of cooperation, migration and global trust across different values of the cost-to-benefit ratio, *r*. The number of tags |*T*| = 2, $\;\hat{\phi }=0.5$, $\;\hat{\theta }=0.5$, the initial size of all groups is 10, and the PGT method is used by the agents to select their migration destination; ‘cooperators’ and ‘migrators’ refer to the proportions of agents who cooperated and migrated, respectively, in the timestep; ‘trust’ refers to the average of the trust of all agents in their group in the timestep. These results show high levels of cooperation being retained for all levels of *r*, and an inverse relation between migration and trust.

Figure 1 shows the percentage of cooperators, migrators and the level of global trust in the population at the end of the simulations. The global trust is the average of the trust of all agents in their group. The results show that we were able to promote and retain high levels of cooperation for all levels of *r* (>65% agents were cooperators). There was only a ≈10% reduction in the level of cooperation as the game difficulty progressively increased from *r* = 0 to *r* = 0.9.

We can also observe from figure 1 an inverse relationship between the levels of trust in the system and the percentage of the population that were migrating at each timestep. This is expected, since agents decide to seek migration to other groups when their trust in their group falls below the trust threshold, $\hat{\phi }$. System-wide trust and migration levels were almost constant from *r* = 0 to *r* = 0.3; there was a sharp change for *r* = [0.4, 0.7], and both values stabilized when *r* ≥ 0.7. As the game difficulty increased, global trust fell from greater than 0.6 to ≈0.38. Similarly, whereas less than 15% of the population were migrating when the game was easiest, more than 35% of them were migrating when the game was the most difficult. Based on this observation, from this point onwards, we will only analyse the level of trust in the system, knowing that the level of migration would be inversely proportional.

The above results also show a weak correlation between the level of global trust and cooperation in the system. The correlation is stronger when the game is easier, but as the game difficulty increases, the correlation is weaker. The results also indicate that, as cooperation decreases, more agents look to migrate.

#### 3.2.1. Impact of trust thresholds

**Figure 2.**
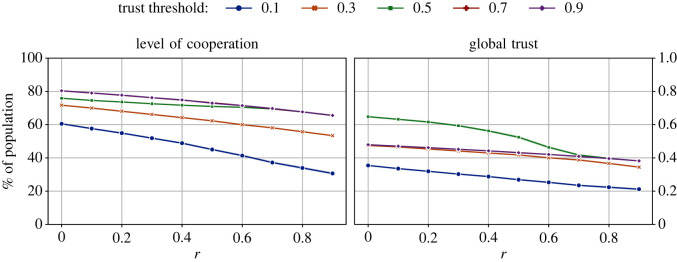
The impact of different values of $\hat{\phi }$ on the levels of cooperation and global trust across different values of the cost-to-benefit ratio, *r*. The number of tags |*T*| = 2 and the initial size of all groups is 10; $\hat{\phi }$ means the trust threshold before an agent decides to seek migration. These plots indicate that higher values of $\hat{\phi }$ are beneficial for cooperation, and that a central value of $\hat{\phi }$ is ideal for promoting global trust.

We conducted further experiments to analyse the impact of different trust thresholds. Figure 2 shows different levels of cooperation and global trust obtained with different values of trust thresholds.

The results in figure 2 show that higher thresholds lead to higher levels of cooperation; $\hat{\phi }=0.1$ provided only ≈60% cooperation when the game was easiest, whereas this was ≈80% for *th* = 0.7. $\hat{\phi }=0.5$ and $\hat{\phi }=0.7$ provided the best results, with $\hat{\phi }=0.7$ doing slightly better. However, the difference between the levels of cooperation seen with the two values gradually disappeared as *r* increased (i.e. the game became more difficult). Similarly, we can see that $\hat{\phi }=0.5$ promoted the highest level of global trust. When $\hat{\phi }$ was too low (0.1), global trust was the lowest. However, an interesting observation is that when $\hat{\phi }=0.3$ or 0.7, i.e. equidistant from 0.5, the global trust was almost identical. We can conclude from these results that a central value of trust threshold is ideal for promoting global trust.

#### 3.2.2. Impact of group sizes

**Figure 3.**
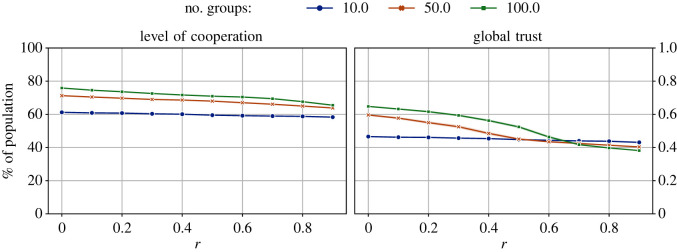
The impact of different values of initial group sizes on the levels of cooperation and global trust across different values of the cost-to-benefit ratio, *r*. The number of tags |*T*| = 2, $\hat{\phi }$ = 0.5, and the initial size of all groups is 10. Overall, these results show that smaller group sizes (more groups) lead to higher levels of cooperation and global trust.

We repeated the above experiments with two more initial group sizes: 50 and 100. Given that the total population is always 1000, these group sizes correspond to 20 and 10 groups in the population, respectively. While the population of groups can fluctuate due to migration, the number of groups will remain constant (see §2.5). Figure 3 shows the levels of cooperation and trust obtained with different initial group sizes.

The results in figure 3 show that when there are more groups (i.e. smaller group sizes), higher levels of cooperation and trust can be achieved. However, even with only 10 groups (a relatively large group size of 100), we had >60% cooperators in the population. Regarding trust, as the game became more difficult, global trust declined, except in the case of 10 groups. There were only marginal differences in the levels of trust in the system for all initial group sizes when the game was most difficult. As the game gets more difficult, more agents migrate, which leads to a decline in the trust (see §3.2). With more options available with smaller groups (i.e. more groups), this reversal is expected.

### 3.3. Migration decisions

In this section, we analyse two migration related decisions and their impact on the levels of cooperation and trust in the system. The first decision determines how long an agent has to wait before migrating again, and the second decision is related to the selection of the migration destination. These are discussed in detail in the following subsections.

#### 3.3.1. Impact of waiting before migrating again

**Figure 4.**
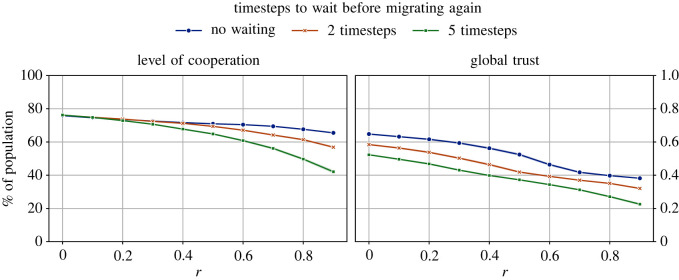
The impact of waiting (or not) before migrating after having migrated in the previous timestep, across different values of the cost-to-benefit ratio, *r*. The number of tags |*T*| = 2 and the initial size of all groups is 10; $\hat{\phi }$ means the trust threshold before an agent decides to seek migration. These results indicate that, overall, waiting has a negative impact on both the levels of cooperation and global trust.

In the experiments reported so far, no restrictions were placed on how soon agents were able to migrate again after the previous migration. Here, we study if asking agents to wait for a while before migrating again might have any impact on the levels of cooperation or trust in the system. We compared the earlier results with those obtained by forcing agents to wait for at least two or five timesteps before they were allowed to migrate again. The results of these comparisons can be found in figure 4.

We see from figure 4 that, when *r* was low, the wait period had almost no impact on the level of cooperation compared to not waiting before migrating again. As the game difficulty increased, having to wait for two timesteps before migrating led to lower levels of cooperation. The difference is more noticeable in the case of global trust, where not having to wait before migrating again leads to higher global trust.

#### 3.3.2. Migration destination selection methods

All agents decide to seek migration if their trust in their group is below the trust threshold. Once the decision has been made, we have so far used only one method (PGT) to select the migration destination. In this section, we analyse the impact of using different destination selection methods on both the levels of cooperation and trust in the system. We compare the three destination selection methods described in §2.5.

**Figure 5.**
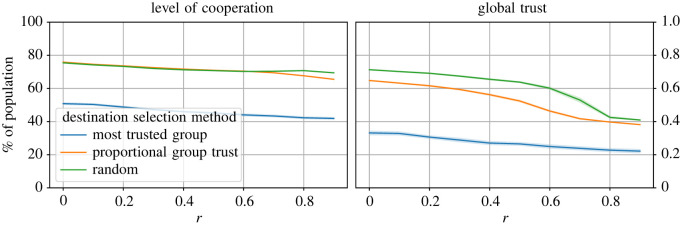
The levels of cooperation and trust in the system obtained with three different destination selection methods, across different values of the cost-to-benefit ratio, *r*. The number of tags |*T*| = 2, $\hat{\phi }=0.5$, $\hat{\theta }=0.5$, and the initial size of all groups is 10. These results show that selecting the most trusted group as the migration destination leads to significantly low levels of cooperation and global trust compared to the other two methods.

The levels of cooperation and trust in the system obtained with different destination selection methods are shown in figure 5. Generally, there is no difference in the level of cooperation obtained by the RDS and PGT methods: they mostly provided >70% cooperation even as the game difficulty increased. The MTG method did not perform very well, however, and the level of cooperation ranged between 41 and 50% for different levels of game difficulty. The global trust obtained with the most trusted group method was similarly significantly less than that achieved with proportional group trust and random methods.

**Figure 6.**
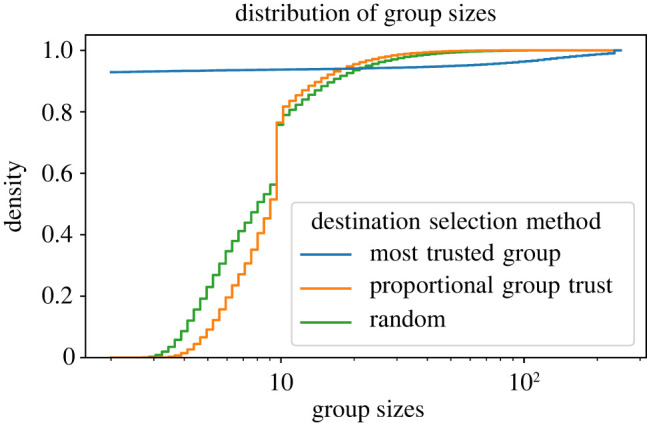
The cumulative distribution of group sizes for three destination selection methods averaged over 50 MC runs and all levels of game difficulty. The number of tags |*T*| = 2, $\hat{\phi }=0.5$, $\hat{\theta }=0.5$, and the initial size of all groups is 10. The plot shows that always selecting the most trusted group as the migration destination leads to most agents being concentrated in a few very large groups, whereas the other two methods lead to an even distribution of group sizes.

We suspected that the MTG method did not perform as well because the population might have evolved to have some large groups and many very small groups, situations where the action is a coin toss. We analysed the distribution of group sizes for all three methods, and the results are plotted in figure 6. The results show that, with the MTG method, most groups had the minimum number of agents (2), since most agents migrated to a few groups that were regarded as ‘most trusted’ neighbouring groups. However, in the case of the PGT and RDS methods, most group sizes were close to the mean group size (10), and only a few were large groups. Inferring to levels of cooperation obtained with these methods, it appears that a more uniform distribution of group sizes, or a uniform distribution of agents in groups within the population, is more conducive to the promotion of cooperation.

Both RDS and PGT methods randomly select a destination group, but PGT is more restrictive because the trust in the destination group has to be more than the current group. Since agents seek migration only when they do not have enough trust in the current group, not allowing agents to migrate immediately leads to lower trust.

These observations are in line with the earlier results that showed that disallowing migration immediately after migrating in the previous timestep led to deterioration in the trust (see §3.3.1). The level of trust is the lowest with the MTG method. Even though agents always migrate once they have decided to seek migration with this method, as discussed above (figure 4), this leads to many very small groups and a few large groups. It appears that this skewed distribution is not conducive to promoting trust in the system.

Therefore, we can conclude from the above discussion that two conditions are required for promoting high levels of cooperation and trust in the system: agents should be allowed to migrate once they do not have enough trust in their group, and the destination selection method should be able to maintain a relatively uniform distribution of group sizes.

### 3.4. Impact of having different number of tags

**Figure 7.**
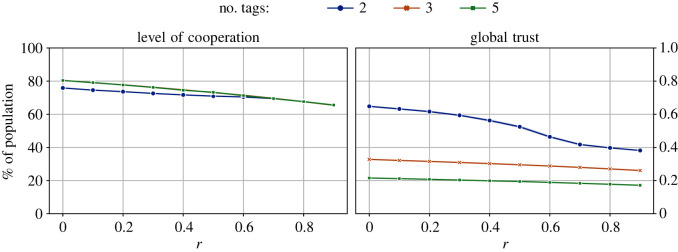
The impact of the number of tags on the levels of cooperation and trust in the system across different values of the cost-to-benefit ratio, *r*. The initial size of all groups is 10, and the values of both cooperation and trust threshold are 0.5. Overall, the plots indicate minimal impact of the number of tags on cooperation; global trust, though, is more when there are fewer tags.

We conducted experiments with different numbers of tags |*T*| = {2, 5, 10} to study if the number of tags has any impact on both the level of cooperation and trust in the system. The results for these experiments are shown in figure 7. From the figure, we see that the number of tags has a minimal impact on the level of cooperation, whereas its impact on the level of global trust is significant. The higher the number of tags in the system, the lower the overall system trust.

**Figure 8.**
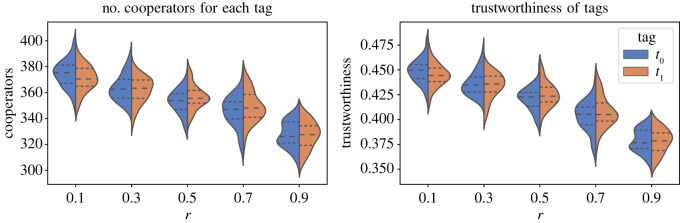
Level and distribution of the percentage of the number of cooperators and the level of trustworthiness of each tag in the population. The initial size of all groups is 10, the values of both cooperation and trust threshold are 0.5, and the results are averages over all values of *r*. These results show that tags evolve to have different levels of cooperation and trustworthiness.

**Figure 9.**
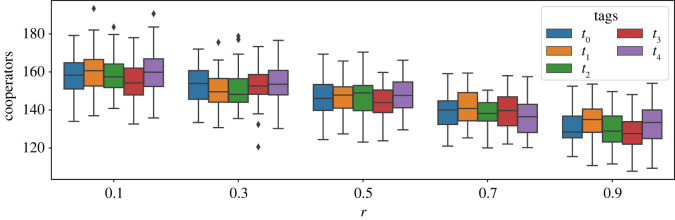
Boxplots of the levels of cooperation for all five tags across all values of *r* (black diamonds indicate outliers to the results shown). Values of both cost and trust threshold are 0.5, and the initial group size is 10. These plots indicate that tags eventually evolve to have different levels of cooperation.

We further analysed the results to check if different tags can evolve to have different levels of cooperation, or if different tags were trusted differently by the agents in the population. Figure 8 shows violin plots for the levels of cooperation and trustworthiness of different tags, for the simulation with two tags. We can see from the results that the two tags had different levels of cooperation and trustworthiness for all levels of game difficulty. While these differences might be minor, the observations are consistent in all cases and indicate that the tags always evolve to have different levels of cooperation and trustworthiness. We also repeated the above analysis for five tags to corroborate our intuition that tags evolve to have different levels of cooperation. Figure 9 shows the distribution of the levels of cooperation for all five tags for all levels of game difficulty. These results also show that all tags had diverse levels of cooperation. Therefore, we can conclude that the tags evolve to have different levels of cooperation and trust in the simulation.

### 3.5. Relationship between groups and tags

The final study we did was to check if agents with a particular tag would be concentrated in a particular group, or if they would be distributed evenly across different groups. Agents do not have any preference for groups with their own tags when they migrate. The decision is made based on how much they trust that group. In order to find out if agents with the same tag were congregating within certain groups, we analysed the percentage of a tag’s total population across all groups at the end of the simulation.

**Figure 10.**
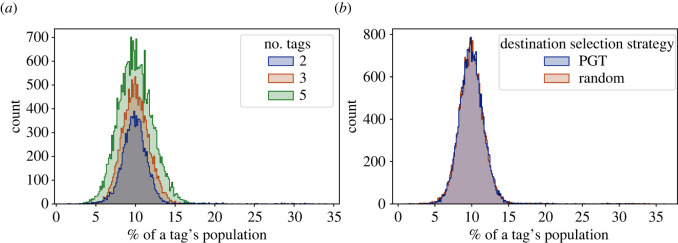
The number of groups versus the percentage of a tag’s population in that group at the end of the simulation for (*a*) different number of tags and (*b*) destination selection methods. The initial group size is 10, the migration wait period is 0, and the trust threshold is 0.5. Both plots show that the population of all tags is evenly spread across all groups.

Figure 10 shows a histogram plot for the percentage of a tag’s total population found in each group when the number of groups was 10, trust threshold was 0.5, migration wait period was 0 and the PGT destination selection method was used. We can see from the figure that a vast majority of any tag’s population within a group was in the range 8–12%, meaning that the tag population was evenly spread across the groups. This was true when comparing either different number of tags or the two destination selection strategies: PGT and RDS. Note that the MTG destination selection strategy was not used in these comparisons because, as discussed in §3.3.2, with MTG, the population would be distributed into many very small groups and a few very large groups.

**Figure 11.**
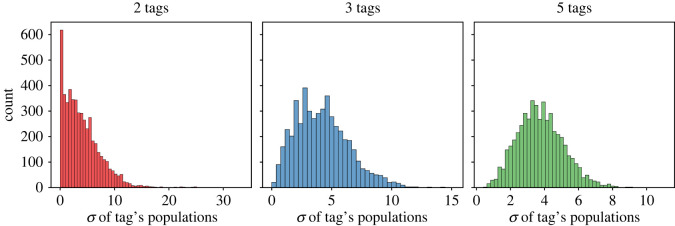
Standard deviations of different tags’ population within groups. The initial group size is 10, the migration wait period is 0, and the trust threshold is 0.5. All plots indicate that, overall, the population of each tag is evenly spread across different groups.

We also analysed the distribution of different tags within each group. Figure 11 shows a histogram plot of the standard deviations of all tags’ populations within each groups, for simulations with different numbers of tags (2, 3, 5). We can see from the plots that all tags were generally evenly distributed within different groups. Based on this information and that in the previous paragraph, we can conclude that, in our simulations, agents with different tags do not congregate in certain physical groups; they are uniformly distributed across all groups.

## 4. Conclusion

In this paper, we presented an N-player evolutionary game model that integrates tags, trust and migration to study how trust in our social and physical groups might influence our cooperation and migration decisions. Our model is able to promote and retain high levels of cooperation in almost all cases of the simulation. High levels of trust are also obtained under various game settings, though this level varied more than the levels of cooperation. The results indicate that the integration of trust and tags, together with controlled migration, is generally conducive to promoting high levels of cooperation among the population.

We observed that curbing the migration decisions of agents, either through destination selection methods or by asking them to wait for a while before migrating again, can impact both the levels of cooperation and trust in the population. As the game became more difficult, making agents wait before migrating again led to significant drops in cooperation. This suggests that allowing agents to seek better options through migration is an important factor for promoting cooperation and trust. Similarly, having a more uniform distribution of group sizes, i.e. fewer extremes in terms of group sizes, through more balanced destination selection methods also led to higher levels of cooperation and trust in the population.

In the simulation, an agent’s actions were not determined by its tag: however, we observed that tags evolved to have different levels and distributions of cooperators and trustworthiness. This shows that even if we create artificial labels and tags in a society, over time those labels might evolve to have different attributes as a whole.

The number of different tags in the system does not impact the levels of cooperation, but impacts the level of trust. The model has no explicit relationship between tags and cooperation and no relationship evolved during the simulation. As for the overall trust in the system, it is easier to promote trust in tags when there are fewer of them. When there are more tags, most probably due to the diversity in the system, it becomes difficult to promote trust. Finally, we also observed that all tags are generally uniformly spread across different groups, and there were only minor variations in the diversity of any group’s populations in terms of different tags.

In our current model, all agents use the same destination selection method and have the same level of trust threshold that determines when they seek migration. They also use the same method to determine their cooperation/defection actions. In the future, we plan to study how the model would evolve when there are variations between individuals within the population [42], for example, when there are different methods and destination selection methods competing with each other in the population, or when agents have varying degrees of trust thresholds. Moral preferences, both personal and social, have recently been proposed for explaining unselfish behaviour [1]. We also plan to explore how the diffusion of such preferences among interconnected networks through migration might impact the evolution of cooperation and trust in those networks of individuals.

## Data Availability

Data and relevant code for this research work are stored in GitHub: https://github.com/sandeepdhakal/abm-trust and have been archived within the Zenodo repository https://doi.org/10.5281/zenodo.6409293.

## References

[1] Capraro V, Perc M. 2021 Mathematical foundations of moral preferences. J. R. Soc. Interface **18**, 20200880. (10.1098/rsif.2020.0880) 33561377 PMC8086879

[2] Nowak MA. 2006 Five rules for the evolution of cooperation. Science **314**, 1560-1563. (10.1126/science.1133755) 17158317 PMC3279745

[3] Dong R, Jia X, Wang X, Chen Y. 2020 Optimal tag-based cooperation control for the ‘prisoner’s dilemma’. Complexity **2020**, 1-19. (10.1155/2020/8498613)

[4] Hu Z, Li X, Wang J, Xia C, Wang Z, Perc M. 2021 Adaptive reputation promotes trust in social networks. IEEE Trans. Netw. Sci. Eng. **8**, 3087-3098. (10.1109/TNSE.2021.3103771)

[5] Kumar A, Capraro V, Perc M. 2020 The evolution of trust and trustworthiness. J. R. Soc. Interface **17**, 20200491. (10.1098/rsif.2020.0491) 32781937 PMC7482572

[6] Yucel O, Crawford C, Sen S. 2015 Evolving effective behaviours to interact with tag-based populations. Connect. Sci. **27**, 288-304. (10.1080/09540091.2015.1031467)

[7] McCauley CR, Jussim LJ, Lee YT. 1995 Stereotype accuracy: toward appreciating group differences. In *Stereotype accuracy: toward appreciating group differences* (eds YT Lee, LJ Jussim, CR McCauley), pp. 293–312. Washington, DC: American Psychological Association.

[8] Kim JW. 2010 A tag-based evolutionary prisoner’s dilemma game on networks with different topologies. J. Artif. Soc. Soc. Simul. **13**, 2. (10.18564/jasss.1584)

[9] Greenwood GW. 2011 Enhanced cooperation in the N-person iterated snowdrift game through tag mediation. In *2011 IEEE Conf. on Computational Intelligence and Games, (CIG 2011)*, pp. 1–8.

[10] Howley E, Duggan J. 2010 The evolution of cooperation and investment strategies in a commons dilemma. In *Proc. of the Adaptive and Learning Agents Workshop, ALA 2010 - In Conjunction with the 9th Int. Conf. on Autonomous Agents and Multiagent Systems*, pp. 93–99. AAMAS 2010.

[11] Hadzibeganovic T, Lima FWS, Stauffer D. 2012 Evolution of tag-mediated altruistic behavior in one-shot encounters on large-scale complex networks. Comput. Phys. Commun. **183**, 2315-2321. (10.1016/j.cpc.2012.05.020)

[12] Drouvelis M, Grosskopf B. 2021 The impact of smiling cues on social cooperation. South. Econ. J. **87**, 1390-1404. (10.1002/soej.12485)

[13] Tian L, Li M, Yu H, Jin X. 2017 Attribute-based partner updating boosts cooperation in social P2P systems. In *2017 IEEE 15th Int. Conf. on Dependable, Autonomic and Secure Computing, 15th Int. Conf. on Pervasive Intelligence and Computing, 3rd Int. Conf. on Big Data Intelligence and Computing and Cyber Science and Technology Congress (DASC/PiCom/DataCom/CyberSciTech), Orlando, FL, 6–10 November 2017*, pp. 1226–1231. New York, NY: IEEE.

[14] Skyrms B, Pemantle R. 2009 A dynamic model of social network formation. In *Understanding complex systems* (eds T Gross, H Sayama), pp. 231–251. Berlin, Germany: Springer. 10.1073/pnas.97.16.9340 PMC16869 10922082

[15] Santos FC, Pacheco JM, Lenaerts T. 2006 Cooperation prevails when individuals adjust their social ties. PLoS Comput. Biol. **2**, e140. (10.1371/journal.pcbi.0020140) 17054392 PMC1617133

[16] Dhakal S, Chiong R, Chica M, Middleton RH. 2020 Climate change induced migration and the evolution of cooperation. Appl. Math. Comput. **377**, 125090. (10.1016/j.amc.2020.125090)

[17] Buesser P, Tomassini M, Antonioni A. 2013 Opportunistic migration in spatial evolutionary games. Phys. Rev. E **88**, 042806. (10.1103/PhysRevE.88.042806) 24229225

[18] Wu T, Fu F, Zhang Y, Wang L. 2012 Expectation-driven migration promotes cooperation by group interactions. Phys. Rev. E **85**, 066104. (10.1103/PhysRevE.85.066104) 23005159

[19] Barreira da Silva Rocha A. 2013 Evolutionary dynamics of nationalism and migration. Physica A **392**, 3183-3197. (10.1016/j.physa.2013.03.030)

[20] Cardinot M, O’Riordan C, Griffith J, Szolnoki A. 2019 Mobility restores the mechanism which supports cooperation in the voluntary prisoner’s dilemma game. New J. Phys. **21**, 073038. (10.1088/1367-2630/ab3064)

[21] Boyd R, Richerson PJ. 2009 Voting with your feet: payoff biased migration and the evolution of group beneficial behavior. J. Theor. Biol. **257**, 331-339. (10.1016/j.jtbi.2008.12.007) 19135062

[22] Chica M, Chiong R, Kirley M, Ishibuchi H. 2018 A networked *N*-player trust game and its evolutionary dynamics. IEEE Trans. Evol. Comput. **22**, 866-878. (10.1109/TEVC.2017.2769081)

[23] Chica M, Chiong R, Ramasco JJ, Abbass H. 2019 Effects of update rules on networked N-player trust game dynamics. Commun. Nonlinear Sci. Numer. Simul. **79**, 104870. (10.1016/j.cnsns.2019.104870)

[24] Chica M, Chiong R, Adam MTP, Teubner T. 2019 An evolutionary game model with punishment and protection to promote trust in the sharing economy. Sci. Rep. **9**, 19789. (10.1038/s41598-019-55384-4) PMC6930269 31874960

[25] Chiong R, Dhakal S, Chaston T, Chica M. 2022 Evolution of trust in the sharing economy with fixed provider and consumer roles under different host network structures. Knowledge-Based Syst. **236**, 107496. (10.1016/j.knosys.2021.107496)

[26] Hadzibeganovic T, Xia C. 2016 Cooperation and strategy coexistence in a tag-based multi-agent system with contingent mobility. Knowledge-Based Syst. **112**, 1-13. (10.1016/j.knosys.2016.08.024)

[27] Jensen GG, Tischel F, Bornholdt S. 2019 Discrimination emerging through spontaneous symmetry breaking in a spatial prisoner’s dilemma model with multiple labels. Phys. Rev. E **100**, 062302. (10.1103/PhysRevE.100.062302) 31962486

[28] Cohen E, Haun D. 2013 The development of tag-based cooperation via a socially acquired trait. Evol. Hum. Behav. **34**, 230-235. (10.1016/j.evolhumbehav.2013.02.001)

[29] Birk A. 2000 Boosting cooperation by evolving trust. Appl. Artif. Intell. **14**, 769-784. (10.1080/08839510050127542)

[30] Tanimoto J. 2013 Difference of reciprocity effect in two coevolutionary models of presumed two-player and multiplayer games. Phys. Rev. E **87**, 062136. (10.1103/PhysRevE.87.062136) 23848656

[31] Ariful Kabir KM, Tanimotoc J. 2019 Impact of awareness in metapopulation epidemic model to suppress the infected individuals for different graphs. Eur. Phys. J. B **92**, 199. (10.1140/epjb/e2019-90570-7)

[32] Allport GW. 1979 The nature of prejudice, Unabridged, 25th anniversary ed ed., Reading, MA: Addison-Wesley Pub. Co.

[33] Szabó G, Töke C. 1998 Evolutionary prisoner’s dilemma game on a square lattice. Phys. Rev. E **58**, 69-73. (10.1103/PhysRevE.58.69)

[34] Chiong R, Kirley M. 2012 Effects of iterated interactions in multiplayer spatial evolutionary games. IEEE Trans. Evol. Comput. **16**, 537-555. (10.1109/TEVC.2011.2167682)

[35] Wang Z, Kokubo S, Jusup M, Tanimoto J. 2015 Universal scaling for the dilemma strength in evolutionary games. Phys. Life Rev. **14**, 1-30. (10.1016/j.plrev.2015.04.033) 25979121

[36] Ito H, Tanimoto J. 2018 Scaling the phase-planes of social dilemma strengths shows game-class changes in the five rules governing the evolution of cooperation. R. Soc. Open Sci. **5**, 181085. (10.1098/rsos.181085) 30473853 PMC6227953

[37] Souza MO, Pacheco JM, Santos FC. 2009 Evolution of cooperation under N-person snowdrift games. J. Theor. Biol. **260**, 581-588. (10.1016/j.jtbi.2009.07.010) 19616013

[38] Szolnoki A, Perc M, Danku Z. 2008 Towards effective payoffs in the prisoner’s dilemma game on scale-free networks. Physica A **387**, 2075-2082. (10.1016/j.physa.2007.11.021)

[39] Chica M, Rand W. 2017 Building agent-based decision support systems for word-of-mouth programs: a freemium application. J. Mark. Res. **54**, 752-767. (10.1509/jmr.15.0443)

[40] Xiao Z, Chen X, Szolnoki A. 2020 Leaving bads provides better outcome than approaching goods in a social dilemma. New J. Phys. **22**, 023012. (10.1088/1367-2630/ab6a3b)

[41] Albert R, Barabási AL. 2002 Statistical mechanics of complex networks. Rev. Mod. Phys. **74**, 47-97. (10.1103/RevModPhys.74.47)

[42] McNamara JM. 2013 Towards a richer evolutionary game theory. J. R. Soc. Interface **10**, 20130544. (10.1098/rsif.2013.0544) 23966616 PMC3785819

